# From Community to Care: CHWs Advancing Age-Friendly Health in FQHCs

**DOI:** 10.1093/geroni/igaf122.4125

**Published:** 2025-12-31

**Authors:** Cherell Cottrell-Daniels, Joycelyn Lawrence, Kevin Espinoza, Katherine Chung-Bridges, Diego Shmuels, Deborah Gracia, Sweta Tewary

**Affiliations:** Health Choice Network, Miami, Florida, United States; Jessie Trice Community Health System, Miami, Florida, United States; Health Choice Network, Inc., Miami, Florida, United States; Health Choice Network, Miami, Florida, United States; Borinquen Healthcare Center, Inc., Miami-Dade, Florida, United States; Borinquen Healthcare Center, Inc., Miami-Dade, Florida, United States; Jessie Trice Community Health Center, Miami, Florida, United States

## Abstract

**Background:**

The growing older adult population in the U.S. has increased demand for health care professionals with specialized training in geriatrics. Geriatric Workforce Enhancement Programs (GWEPs) across the country are tasked with training Community Health Workers (CHWs) to meet this need. This project integrated CHWs into Age-Friendly Health Systems (AFHS) using 4Ms framework (What matters, Mentation, Medication, Mobility). Goals included assessing changes in CHWs knowledge, intent to use training, and baseline geriatric screenings.

**Methods:**

Twelve CHWs completed a 30-hour in-person geriatric care training covering syndromes, community resources, patient-centered care, and the 4Ms of AFHS. Pre/post surveys captured demographics, satisfaction, and self-rated knowledge. The Wilcoxon Signed-Rank Test assessed changes in knowledge. CHWs documented 4Ms discussion with older adults (65+) using the electronic health record (EHR) unstructured notes section and spreadsheets. Data from 55 chart reviews (Feb- April 2025) were assessed for screening rates and documentation consistency.

**Results:**

CHWs were 73% female, 45% White, with an average age of 40 years. Pre-training, 36% rated their knowledge as moderate; post-training, 91% reported high knowledge and high likelihood of applying information. Knowledge gains were statistically significant (z = -2.23, p = .026). CHWs documentation for Medication, Mobility, and What Matters was 100%; Mentation screening was 87% for depression, 31% for dementia, with full completion of all 4Ms in 31% of encounters.

**Conclusion:**

Findings suggest strong adoption of the 4Ms components, ease of documentation, with opportunities for improvement in dementia screenings and overall 4Ms completion. Strengthening EHR infrastructure is needed to support age-friendly care.

